# Comparison of the fecal, cecal, and mucus microbiome in male and female mice after TNBS-induced colitis

**DOI:** 10.1371/journal.pone.0225079

**Published:** 2019-11-08

**Authors:** Ariangela J. Kozik, Cindy H. Nakatsu, Hyonho Chun, Yava L. Jones-Hall

**Affiliations:** 1 Department of Comparative Pathobiology, Purdue University, West Lafayette, Indiana, United States of America; 2 Interdisciplinary Life Sciences, Purdue University, West Lafayette, Indiana, United States of America; 3 Department of Agronomy, Purdue University, West Lafayette, Indiana, United States of America; 4 Department of Mathematics and Statistics, Boston University, Boston, Massachusetts, United States of America; University of Ottawa, CANADA

## Abstract

Crohn’s Disease and Ulcerative Colitis are chronic, inflammatory conditions of the digestive tract, collectively known as Inflammatory Bowel Disease (IBD). The combined influence of lifestyle factors, genetics, and the gut microbiome contribute to IBD pathogenesis. Studies of the gut microbiome have shown significant differences in its composition between healthy individuals and those with IBD. Due to the high inter-individual microbiome variation seen in humans, mouse models of IBD are often used to investigate potential IBD mechanisms and their interplay between host, microbial, and environmental factors. While fecal samples are the predominant material used for microbial community analysis, they may not be the ideal sample to use for analysis of the microbiome of mice with experimental colitis, such as that induced by 2, 4, 6 trinitrobenzesulfonic acid (TNBS). As TNBS is administered intrarectally to induce colitis and inflammation is confined to the colon in this model, we hypothesized that the microbiome of the colonic mucus would most closely correlate with TNBS colitis severity. Based on our previous research, we also hypothesized that sex would be associated with both disease severity and microbial differences in mice with chronic TNBS colitis. We examined and compared the fecal, cecal content, and colonic mucus microbiota of 8-week old male and female C57BL/6J wild-type mice prior to and after the induction of TNBS colitis via 16S rRNA gene sequencing. We found that the colonic mucus microbiome was more closely correlated with disease severity than were alterations in the fecal and cecal microbiomes. We also found that the microbiomes of the feces, cecum, and mucus were distinct, but found no significant differences that were associated with sex in either compartment. Our findings highlight the importance of sampling colonic mucus in TNBS-induced colitis. Moreover, consideration of the differential impact of sex on the microbiome across mouse strains may be critical for the appropriate application of TNBS colitis models and robust comparisons across studies in the future.

## Introduction

Crohn’s Disease (CD), subtype of Inflammatory Bowel Disease (IBD), can affect any region of the GI tract. Although the exact cause of CD is unknown, microbial dysbiosis, genetic susceptibility and environmental factors have been associated with the development and progression of disease [1–4]. The gastrointestinal microbiota has been implicated in many inflammatory diseases, but its specific role is not completely understood. Importantly, there is also a need for studies that examine the microbial communities of different niches within the gastrointestinal tract and how they contribute to or correlate with colitis, particularly in mouse models in which inflammation is often isolated to certain areas of the intestine. Such questions are of clinical importance because of the differences in spatial manifestations of CD and Ulcerative Colitis (UC), and the variable success of microbiome-targeted therapies (such as fecal microbiota transplantation, or FMT) between IBD subtypes[5,6]. Currently, human studies of IBD may consist of any combination of samples representing a variety of niches; fecal samples (luminal content), colonic biopsies (mucosa), and rectal swabs (colorectal mucus layer)[7]. Mucosal samples derived from biopsied diseased tissues are useful for surveying the surfaces involved in active inflammation and sampling from adjacent healthy mucosa allows for direct comparisons between healthy and inflamed microenvironments[8–11]. However, due to the higher costs and invasive nature of biopsying patients, the use of fecal samples is more common[12]. Importantly; however, humans typically have high levels of inter-individual microbiome variation that make it difficult to uncover clear trends without very large sample sizes that can be cost-prohibitive[13–16]. This was confirmed in a recent longitudinal multi-omic study of human IBD that produced evidence of distinct functional dysbiosis and microbial differences associated with IBD phenotype and activity[16]. Unraveling these interactions will likely be key to understanding the role of the microbiome in IBD. However, additional targeted studies are necessary to investigate these relationships. Therefore, mouse models of IBD, such as 2,4,6 trinitrobenzene sulfonic acid (TNBS) induced colitis, remain useful tools to study the interactions between inflammation and the gut microbiome in different niches.

Mouse models of IBD provide access to a similar variety of sample types studied in humans. Fecal samples are widely used in mouse studies to survey the microbiome and its relationship to IBD[17]. Notably, however, the inflammation induced by TNBS colitis is limited to the colon in mice. Therefore, in mice, the information gained from sampling directly at the site of inflammation, such as a colonic mucus sample, could provide a more accurate reflection of microbial activity during inflammation than fecal samples alone. Additionally, data suggests that the mouse mucus layer is a distinct niche with a distinct microbiome[18]. Recent studies of TNBS colitis in mice report a link between the intestinal mucus layer, its microbiota, and inflammation. Microbes in the mucus layer have more direct and sustained contact with the immune system and the intestinal epithelium [19]. TNBS colitis results in decreased fecal microbiota diversity[20], as well as reduced epithelial barrier integrity and altered epithelial responsiveness[21]. In a recent study, similar to our study here, Wardill et al. surveyed the fecal and colonic-adherent microbiota during acute and reactivated TNBS colitis and showed that the colonic-adherent microbiota was more impacted by TNBS colitis than the luminal (fecal) microbiota[22]. Interestingly, despite similar microbiota alterations between acute and reactivated colitis, they observed reduced inflammatory damage and the induction of immune tolerance in response to reactivated colitis[22]. Additionally, another recent study found that TNBS colitis resulted in altered fecal microbiota, but also reduced goblet cells and reduced thicknessof both layers of mucus[23]. Taken together, there is evidence that investigating microbial communities of the mucus layer, in addition to the commonly used fecal sampling, may provide additional information that is key to our understanding of the mechanisms of how the microbiome and colitis intersect.

At the time of writing, we were not aware of other studies investigating the spatial organization of the microbiome in the TNBS mouse model of chronic colitis, nor sex-associated microbial patterns in this model. Previous studies in our lab have determined that sustained production of tumor necrosis factor (TNF) and other host factors, such as sex and age, impact the fecal microbiomes of B6.129S mice with acute TNBS colitis [24,25]. However, CD is a chronic disease, and it is therefore important to also investigate the microbiome in chronic disease in order to determine the effect of long-term inflammation. In these current studies of chronic colitis, we used a different strain of mouse than was used in our previous acute colitis studies. The B6.129S mouse strain is more susceptible to TNBS colitis than is the C57BL/6J mouse stain. C57BL/6J mice were therefore selected for these studies instead of B6.129S for their ability to withstand the 5 weeks of TNBS treatment, which is used to induce chronic colitis. Here, we use the established chronic TNBS model[26] in C57BL/6J male and female mice to evaluate microbiome changes in different niches (feces, cecum and mucus) that are induced by chronic inflammation and determine which community is most closely correlated with colitis. We hypothesized that the microbiome of the colonic mucus would most closely correlate with TNBS colitis. Our hypothesis on sex was that male mice would have more severe colitis than female mice[25] and that sex would be associated with the fecal, cecal, and mucus microbiomes of mice. To test our hypotheses, we collected fecal samples for Illumina MiSeq sequencing before and after chronic TNBS colitis induction in C57BL/6J mice, as well as cecal content and colonic mucus at necropsy to determine the composition and microbial diversity of the cecum and colonic mucus and evaluated colitis histologically.

## Methods

### Mice

All colitis experiments with animals in this study were approved by the Purdue Animal Care and Use Committee (PACUC Protocol #1210000747). Male and female C57BL/6J mice were obtained from The Jackson Laboratory (Bar Harbor, ME). Mice were subsequently bred and housed at Purdue University for at least two generations before mice were selected for these experiments. Mice were housed in specific pathogen free conditions and maintained on 12hr light/dark cycles with free access to food and water. Mice were fed with 2018S mouse chow (Envigo). Mice were caged separately by sex and treatment: TNBS vs SHAM (control). Mice were 8–9 weeks old at the start of each experiment. Mice are described in the figures and text by their assigned treatment group (TNBS or SHAM), however all mice at day 0 are ‘untreated’, thus mice from the TNBS group at day 0 are referred to as ‘pre-colitis’.

### Chronic TNBS colitis

Power calculation determined that at least three mice per treatment group were needed. The experiments were performed three times with at least three mice in each treatment group. TNBS colitis was induced via intra-rectal injections as previously described [24], Briefly, 100 μl of the TNBS intra-rectal (IR) solution (1 volume of 5% w/v TNBS solution mixed with 1 volume of absolute ethanol) was slowly instilled into the lumen of the colon of anesthetized mice. The injections were repeated four times, one injection each week, for a total of five injections. Mice were fasted from food for 24 hours before each injection and were weighed twice per week. Control mice (SHAM) received intra-rectal injections of PBS. Three days after the 5^th^ intra-rectal (IR) injection, necropsy was performed and tissues were harvested. Mice were euthanized by an overdose of CO_2_ followed by cervical dislocation. Fecal samples for microbial community analyses were collected on days 0, 10, and necropsy and immediately frozen and stored at -80°C until analyzed. The cecum was resected, weighed, and its contents collected for sequencing. A section of the distal colon was collected in formalin for histological analysis. Mucus was collected from the remaining colon tissue by gentle scraping of the mucus from the surface of the colon. The mucus was immediately frozen, and stored at -80°C for future sequencing.

### Histological assessment of colitis

Colon tissue was sampled and colitis was assessed as described previously [24,25]. Briefly, the colons were removed, opened and washed with PBS. A portion of distal colon was fixed in formalin for histological examination. Fixed colon sections were stained with hematoxylin and eosin and assessed for severity of colitis using a semi-quantitative rubric (S1 Table). Colitis was assessed and scored by a board certified veterinary pathologist, experienced with assessing mouse intestinal pathology and blinded to the treatment groups using a published semiquantitative assessment method that is used for luminal antigens that induce inflammation[24,27].

### DNA extraction and sequencing

A subset of mice and their samples were randomly selected from all available experimental replicates to form a dataset of 5 male and 5 female mice in each treatment group (TNBS and SHAM) to be sequenced. Total DNA was extracted from each fecal, cecal, and mucus sample using the FastDNA Spin kit for soil (MP Biomedicals), with bead beating, per the manufacturer’s instructions. DNA quality and quantity were assessed as described previously using agarose gels, spectrometry and fluorometry [25]. PCR primers were used to amplify the V3-V4 region of the 16S rRNA gene in DNA from day 0 and fecal, cecal, and mucus DNA from day 38 (necropsy). The resulting amplicons were sequenced using MiSeq Illumina 2x 250 paired end sequencing as described previously [25]. We experienced difficulties in collecting sufficient mucus for sequencing, limiting the number of mucus samples with viable sequence data.

### Sequence processing

Panda software [28] was used to merge high-quality reads after the removal of primer tags and low quality sequence reads. Sequences were analyzed using the QIIME pipeline version 1.9.1 [29]. The “pick open reference OTU” option with default variables and the Greengenes data set (version 13_8) were used to assign taxonomy to the representative OTU sequences. All subsequent comparisons were performed using equivalent numbers of sequence reads (based on the lowest number of sequences obtained from a single sample) per sample that were chosen by rarefaction, unless otherwise noted in the text. Good’s coverage provided an estimate of sequence coverage of the communities used in these analyses. Rarefied analyses of alpha diversity indices (Chao1, observed OTUs, Shannon) were calculated to compare microbiota community diversity within each sample. Beta diversity comparisons among communities were made using the phylogenetic distances unweighted and weighted Unifrac [30] as well as non-phylogenetic distance analysis using Bray Curtis.

### Statistical analysis

All basic statistical analyses were performed using GraphPad Prism version 7.00 for Windows (GraphPad Software, La Jolla California USA). Histological scores were statistically analyzed as described previously [25]. All data in bar graphs or dot plot formats are expressed as mean ± standard error of means (S.E.M.). Statistical significance is p <0.05. Significant differences in alpha diversity were computed with QIIME by pairwise non-parametric t-test with 999 permutations. Significant differences in beta diversity were determined with QIIME by PERMANOVA, and permDISP was used to check for significant differences in dispersion [31,32]. Taxonomic comparisons were conducted with Analysis of Composition of Microbiomes (ANCOM), which utilizes compositional log-ratios to identify statistically significant taxa [33]. Due to low amplicon yield for some of the mucus samples, the mucus data was not rarefied before differential abundance data in order to maintain samples from control animals. These data were instead transformed and tested with ANCOM. Canonical Correspondence Analysis (CCA) [34] was used to determine genera-environment relationships and the interactions between sets of variables in the data. This is a means to understand how the host variables, (such as sample location, mouse strain, sex, age, TNF status, and colitis response) interact and influence the taxa relative abundances. CCA was implemented with the R package “vegan”. CCA model significance (composed of the following variables; sex, GI site, age, colitis severity, and treatment) was tested with ANOVA and step-wise analysis; final model included treatment, GI site, colitis scores, and taxon relative abundance among communities.

## Results

### Sex impacts chronic TNBS colitis

**Fig 1:**
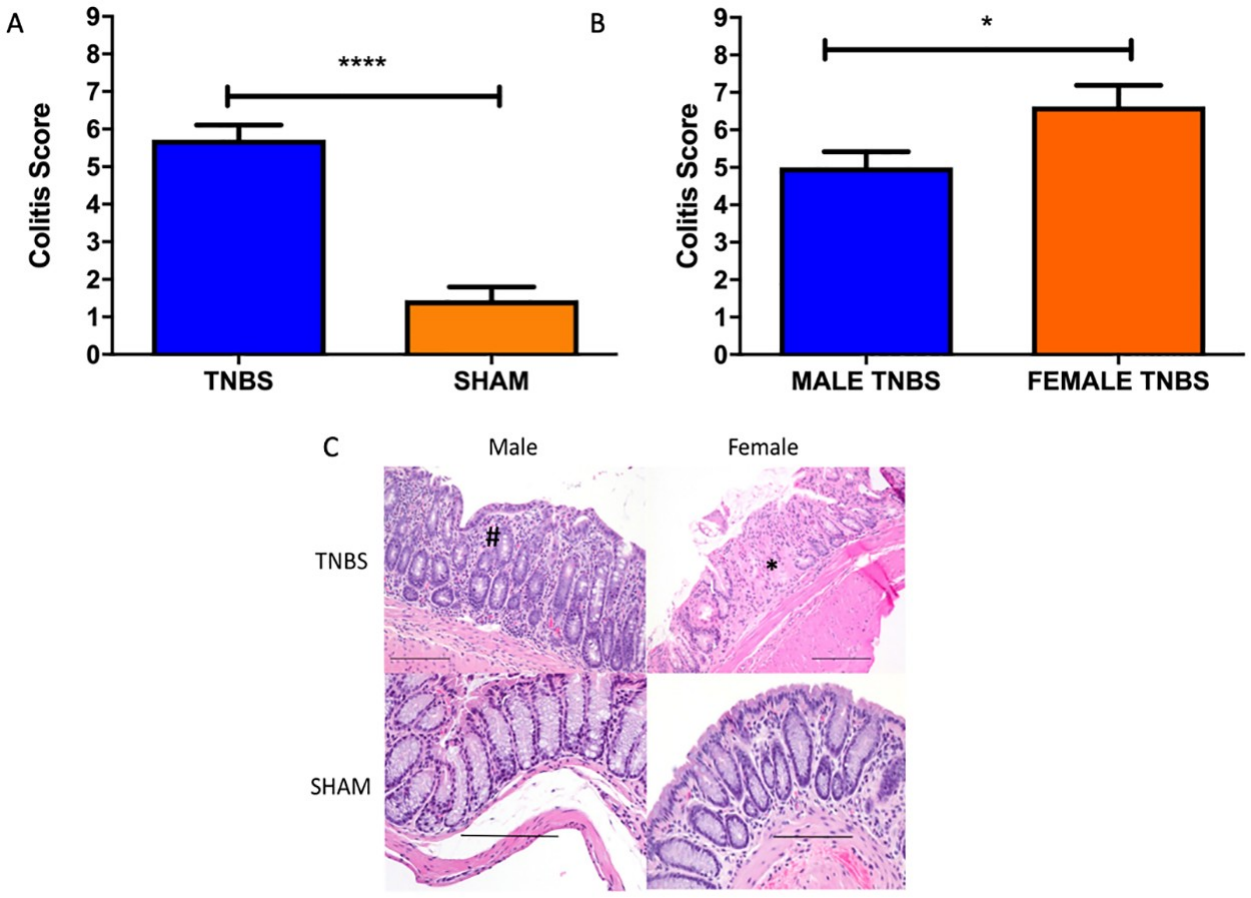
A) Bar Graph colitis scores in TNBS (n = 18) and SHAM (n = 18) treated mice. ****T-test p<0.0001. Values plotted as Mean ± SEM B) Bar graph of differences in colitis score by sex (female n = 8, male n = 10) in TNBS treated mice. T-test *p<0.05. Values plotted as Mean ± SEM C) Hemotoxilin and eosin stained representative photomicrographs of colon showing a mixed inflammatory cell infiltrate in the mucosa of male TNBS treated animals (#) and necrosis and loss of glands, in addition to mild, mixed inflammation (*) in the mucosa of female TNBS treated mice. 20x magnification. Scale bar 100 microns.

Histological evaluation of the colons revealed that overall, TNBS treated mice developed more severe colitis than SHAM treated control mice (Fig 1A), and that, contrary to our acute colitis studies, femaleTNBS treated mice had more severe colitis than male TNBS treated mice (Fig 1B and 1C). There were no significant sex associated differences in the fecal microbiomes of mice pre or post-colitis. There were also no sex associated differences in the cecal microbiomes at necropsy.

### TNBS associated differences in the microbiomes of the feces, cecum, and mucus

**Fig 2:**
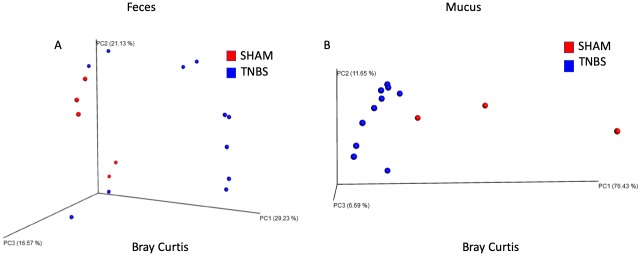
A) Significant differences in beta diversity in the feces of TNBS and SHAM treated mice post-colitis calculated using the Bray Curtis metric. Treatment separates along PC1, which explains approximately 30% of the total variation in the data. Significance found with PERMANOVA p<0.05. PERMDISP indicates dispersion does not contribute to significance. B) Significant differences in beta diversity in the colon mucus of TNBS and SHAM treated mice post-colitis calculated using Weighted UniFrac. Treatment separates along PC1, which explains approximately 76% of the total variation in the data. Significance found with PERMANOVA p<0.05. PERMDISP indicates dispersion does not contribute to significance.

**Fig 3:**
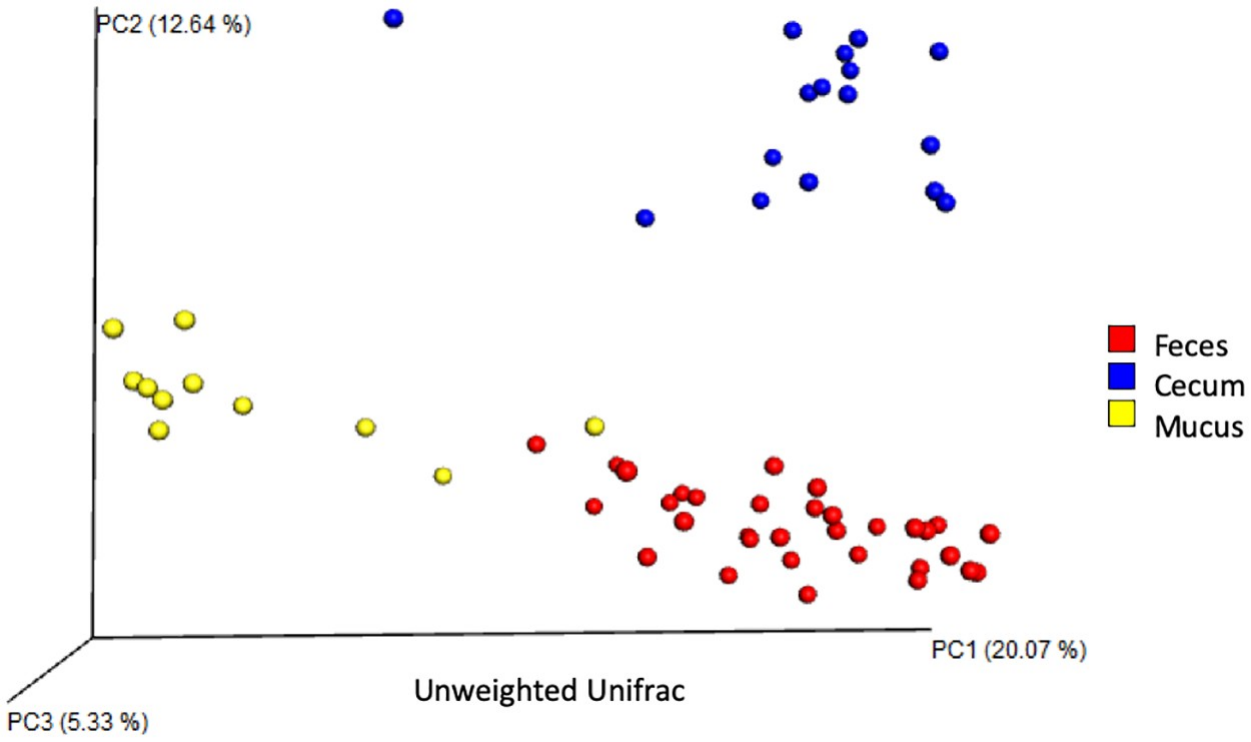
PCoA reveals significant differences in beta diversity by body site, regardless of treatment. Site separates along PC1 and PC2, explaining 20% and 12.6% of the total variation, respectively. Significance found with PERMANOVA (p<0.05). Permdisp indicates that dispersions does not contribute to significance.

Analysis of 7,900 rarefied sequences per sample (Good’s coverage approximately 98%) of TNBS and SHAM treated mice revealed treatment associated differences in beta-diversity (Fig 2). Principal coordinate analysis of the Bray Curtis distance shows that TNBS treatment compared to SHAM resulted in significant differences in beta diversity in the feces (PERMANOVA p<0.05), separating along PC1 and explaining about 30% of the total variation in the data (Fig 2A). PERMDISP indicated that dispersion did not contribute to significance. TNBS treatment also resulted in significant differences in beta-diversity in the mucus (PERMANOVA p<0.05), also separating along PC1 and explaining about 76% of the total variation in the data. (Fig 2B). PERMDISP indicated that dispersion did not contribute to significance. No significant differences in beta diversity in the cecum were associated with TNBS treatment. Sufficient evidence for an impact of TNBS treatment in the feces and the mucus microbiome, but not the cecal microbiome were observed with all three beta diversity metrics (Bray Curtis, weighted UniFrac, unweighted UniFrac) tested. We did not find evidence of treatment associated differences in alpha diversity within the fecal, cecal, or mucus microbiomes. However, principal coordinate analysis of the unweighted UniFrac distance shows that the post-colitis samples cluster by spatial distribution along the GI tract regardless of treatment (Fig 3). The samples separate along PC1, which explains approximately 20% of the total variation in beta-diversity. PERMDISP indicated that dispersion did not contribute to significance, which confirms that the feces, cecum, and mucus have unique microbial communities.

**Fig 4:**
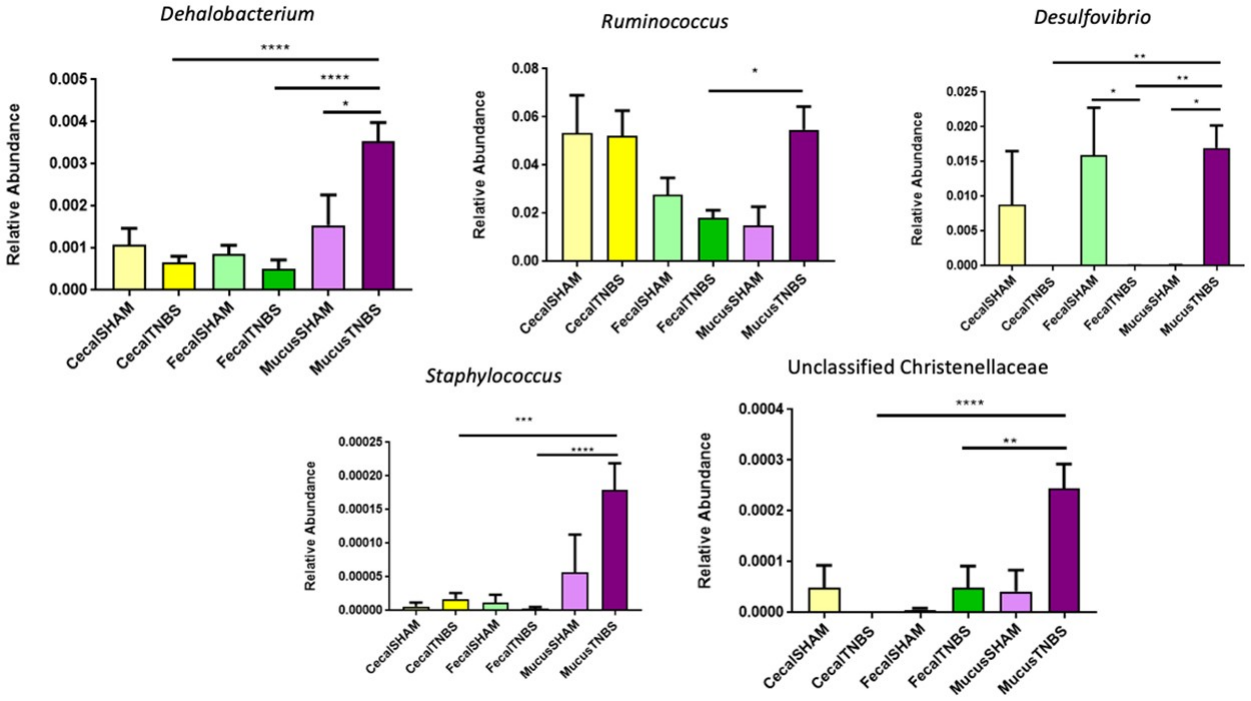
Plots of significantly different taxa across body sites and treatment groups. Overall significance found using ANCOM, and values were corrected for multiple comparisons using False Discovery Rate. Follow-up pairwise tests were performed using Welch’s t-test. *p<0.05, **p < .01, ***p<0.001, ****p<0.0001.

Significant differences in phyla between TNBS and SHAM treated mice across the feces, cecum, and mucus were determined using Kruskal Wallis with follow-up pairwise tests (p-values corrected for multiple comparisons with Dunn’s test). The majority of differences at the phylum level were seen in the mucus samples where the Firmicutes to Bacteroidetes ratio was higher in TNBS compared to SHAM (S1 Fig). ANCOM with false discovery rate (FDR) correction showed significant differences at the genus level associated with treatment at multiple sites (Fig 4). Notably, the mucus of TNBS treated mice had higher relative abundance of *Desulfovibrio* compared to SHAM mice. Additionally, the mucus of TNBS treated mice had the highest relative abundance of *Desulfovibrio* when compared to the cecum and feces of TNBS treated animals. Conversely, the relative abundance of *Desulfovibrio* was significantly lower in the feces of TNBS treated mice when compared to SHAM mice. The mucus of TNBS treated mice also had a significantly higher relative abundance of *Dehalobacterium* compared to SHAM mice. There were also significant differences between the feces, cecum, and mucus of TNBS treated mice. The mucus of TNBS treated mice had a higher relative abundance of *Ruminococcus*, when compared to the feces and a higher relative abundance of *Dehalobacterium*, *Staphylococcus*, and unclassified Christenellaceae than both the cecum and feces of TNBS and SHAM treated mice.

### The mucus microbiome is more closely associated with colitis than is that of the feces or cecum

**Fig 5:**
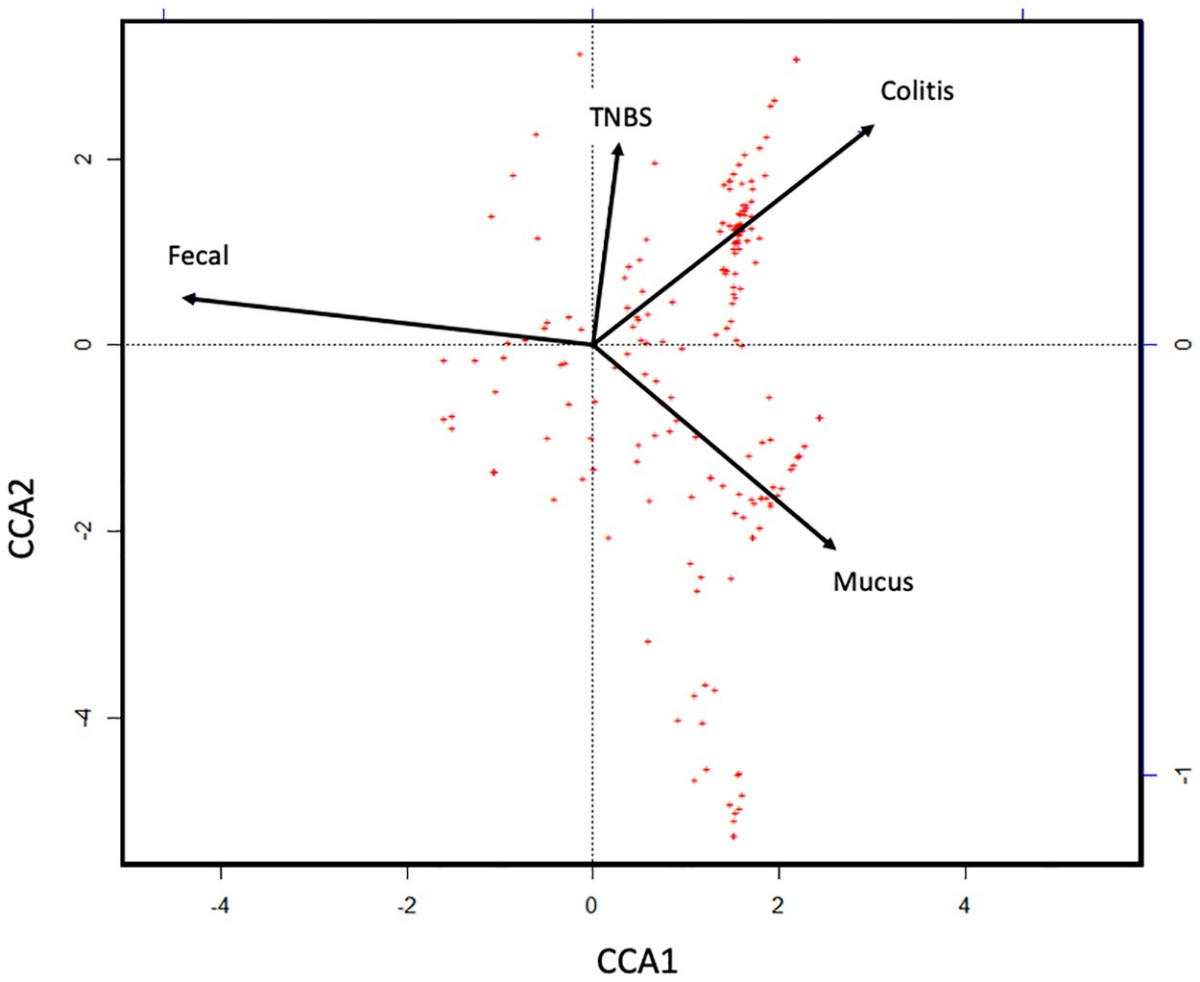
Canonical Correspondence Analysis on the relative taxon abundances reveals that colitis score is more closely associated with the mucus microbiome than the fecal microbiome. Feces is closely associated with CCA1, which explains 52.6% of the constrained variation. TNBS treatment and colitis score was associated with CCA2 and CCA3, which explains 23.8% and 15.2% of the constrained variation, respectively. Significance found using ANOVA p = 0.001.

Canonical Correspondence Analysis of the taxon relative abundances at necropsy show that most of the variability in microbiome abundance is explained by the locations and colitis severity. (Fig 5). TNBS treatment is associated with colitis score, and the mucus appears to be more closely associated with colitis score than the feces. The cecum overlapped with the feces and was excluded from the final model. The fecal sample types were closely associated with CCA1, which explains 52.6% of the constrained variation. TNBS treatment and colitis scores were associated with CCA2 and CCA3 (axis not shown), which explains 23.8% and 15.2% of the constrained variation, respectively.

## Discussion

In order to derive a comprehensive definition of microbal dysbiosis relevant to IBD, microbiome data from many niches along the gastrointestinal tract is required. The objectives of this study were to compare the microbiomes of the feces, cecum, and mucus in mice after chronic TNBS colitis in both male and female mice and to identify microbes at these locations that were significantly altered by chronic TNBS colitis and discover which niche more closely associated with colitis severity. We have shown that the bacterial communities of the fecal content, mucus layer, and cecum of mice, differs at each site. Although all three sites share the same major phyla- Firmicutes, Bacteroidetes, Actinobacteria, and Proteobacteria—there are significant differences at the genus level. Our results here mirror those that have previously been shown in non-treated mice [35–37] and humans [38], and support an understanding of the gastrointestinal regions as microenvironments where the properties of each environment drives taxonomic composition[38].

Our data supports the hypothesis that the mucus environment of mice with chronic TNBS colitis has a distinct microbiota from that of healthy mice and that the microbiota at this location more closely correlates with colitis severity. This is in line with a similar recent work from Wardill, et al., that showed that TNBS colitis has a more substantial effect on the colonic adherent microbiota than the luminal microbiota[22]. The mucus samples revealed the presence of colitis-associated microbes that have been associated with human IBD, although not directly associated with IBD in mice, such as *Ruminococcus* and *Desulfovibrio* [39,40]. Mucus from TNBS treated mice had significantly higher relative abundances of *Desulfovibrio*; a genus of sulfate reducing bacteria (SRB), in the mucus of TNBS treated animals compared to SHAM animals. This finding is consistent with other reports that SRB are increased in the colon of mice with TNBS colitis, associated with more penetrable mucus, and are increased in the mucosa of patients with IBD [39–41]. SRB accentuate the damage caused by TNBS and is associated with increased cytotoxic hydrogen sulfide production and a Th-17 immune response in mice [42,43]. Importantly, production of hydrogen sulfide by SRB is also damaging to the human colon, leading to epithelial damage and inflammation[44]. We also found a significantly higher relative abundance of *Dehalobacterium* in TNBS mucus samples compared to SHAM mucus. While the role of *Dehalobacterium* in IBD is unknown, it has been positively associated with tumorigenesis in a mouse model of colorectal cancer, thus future work could investigate the role of *Dehalobacterium* has in chronic inflammation [45]. There was also significantly higher relative abundance of *Ruminococcus* and *Staphylococcus* in TNBS mucus samples compared to TNBS fecal samples. While *Ruminococcus* is known to perform beneficial functions, such as fermentation [46], some members of *Ruminococcus* are known to degrade mucins in the mucus layer, which provides an energy source for other bacteria [3]. TNBS has been shown to disrupt the colonic epithelial barrier, inhibit colonic motility, and activate innate immune cells[47]. A damaged epithelial barrier and mucus layer could therefore provide an environment for bacteria that are not normally exposed to deeper layers of healthy tissue to colonize, thereby continuing to provide inflammatory stimulus. Wardill et al. show that after initial alterations in the microbiota and immune response due to acute TNBS colitis in mice; reactivated TNBS colitis (at 28 days post-initial exposure) results in similar alterations in microbiota, but a suppressed immune response, and less inflammatory damage[22]. This suggests that the immune responses to bacteria rather than outright impacts of altered microbiota themselves, may play a significant role in colitis. Further longitudinal studies of the interactions between mucus-adherent bacteria (such as SRB), hydrogen sulfide production, and the intestinal mucosa, are needed to define how the microbiota may participate in gut barrier disruption and immune responses.

The results of the CCA suggest that the mucus microbiome is reflective of TNBS colitis activity, perhaps providing more useful information than fecal samples alone. Fecal samples are widely used as surrogates in animal studies to monitor the temporal dynamics of gut microbiomes in relation to disease, and in response to a variety of possible interventions (e.g. nutrition) in the same animal [48]. Despite some debate on the degree of relevance of microbiome results obtained from fecal samples from IBD mouse models in relation to human CD, [49,50] mouse fecal samples are abundant, easy to collect, and remain a cost-effective way to survey the microbiome in models of IBD.

However, human studies often examine patient biopsies from the diseased areas (often at time of diagnosis), to identify taxa associated with active inflammation[51]. Therefore, we suggest that future IBD studies of TNBS colitis should characterize the mucus microbiome in addition to, or instead of, the fecal microbiome.

We also sought to understand how sex impacts the microbiome before and after chronic colitis. Although female mice did have slightly more severe colitis than male mice, there were no sex associated differences in the fecal or cecal microbiomes before or after chronic TNBS colitis in these C57BL/6J mice. This result is in contrast to our other published work in the acute TNBS B6.129S mouse model, where we found that sex differences in the fecal microbiome of mice with acute TNBS colitis correlated with colitis severity [25]. This apparent difference could in fact be due to one or a combination of other host and environmental factors. While housing-related differences in our studies are unlikely (mice housed in same room), a striking study by Jakobsson et al. reported differences in mucus barrier structure and fecal microbiota structure in genetically-identical mice housed in separate rooms[41]. They also demonstrated the ability to produce similar mucus phenotypes by transferring microbiota into germ-free mice.

Differences in our studies could be due to the fact that a different strain of mouse was used in our previous studies (B6.129S mice in the acute, C57BL/6J mice in the chronic) or that the microbiomes of C57BL/6J mice simply do not differ by sex. Sex and strain specific differences in the mouse microbiome have been reported in a recent study. Elderman et al found strain-dependent sex differences in the composition of the gut microbiota, and correlations between the gut microbiota and expression of immune genes in the intestine [52]. There has been conflicting evidence for sex differences in the human microbiome. Most studies reveal little evidence of significant differences in microbial diversity between men and women; however, some studies have found taxa that differ in relative abundance [53–57] and it is suggested that the conflicting reports may be due to overwhelming influence of other factors such as genetics, the reproductive stage in females, and environmental exposures that impact the microbiome.

It has been proposed that microbiome-independent differences in the immune system of male and female mice select for sex-specific microbiome configurations, which can then drive further sex-specific immune responses [58]. There is also evidence of sex-associated differences in immune responses in humans [59–61]. However, these differences are not always associated with the microbiome. A recent study found microbiome-independent sex differences in the B cell development, T-helper cell differentiation, and antigen-presentation pathways in systems of germ free C57BL/6J mice [58]. Therefore, It could be that sex-associated immunological differences (rather than microbiome differences) lead to differing responses to TNBS colitis. This possibility highlights the importance of considering the impact of other variables on the microbiome, especially any sex differences, in the context of colitis. More studies are needed to specifically investigate the role sex plays in mouse models of IBD.

In summary, we have shown that the microbiome of the feces, cecum, and mucus are distinct from one another in this model. Additionally, the impact of TNBS colitis on the microbiome most correlates with the composition of the microbiome in the mucus and the changes that occur in this community are of direct relevance to CD. Our work is unique in that it is one of few recent studies to use a mouse model of chronic TNBS colitis to survey the microbiome in three sites and investigate the impact of sex on the microbiome in the context of disease. Our work illustrates that more extensive sampling, (including baseline mucus samples, sampling from inflamed and normal areas in the same mouse, sampling from different regions of the GI tract, and increased sample size) in colitis studies would likely result in the identification of microbes that are directly impacted by colitis and therefore more relevant to CD. Investigating the spatial variability of the mouse gut microbiome in the context of colitis will allow for a more complete picture of microbial community dynamics during inflammation, and incorporating the mucus microbiome may indentify taxa of interest that warrant further mechanistic investigations. Lastly, while mice will undoubtedly continue to be widely used for microbiome studies, researchers seeking to elucidate the interactions between the microbiome and human CD should consider using animals that have a gastrointestinal physiology that is more similar to humans, such as the pig. Pigs are omnivorous and have digestive processes that are similar to humans [62–64]. They are being increasingly used to model a number of human diseases [63], inflammation [65], as well as, TNBS-induced colitis [66]. The combination of using a model with similar functional anatomy and sampling from sites other than feces (such as mucosal biopsies or mucus) is likely to produce a more accurate picture of the interaction between the microbiome and colitis that is relevant to human CD.

## Supporting information

S1 Fig Taxonomic profile comparisons of phyla in TNBS and SHAM treated samples in each site. Significance indicated by asterisk. Significance found using Kruskal-Wallis with pairwise follow-up tests. P values corrected for multiple comparisons with Dunn’s test. Low abundance phyla plotted individually for visibility.
(TIF)

S1 Table Rubric for semi-quantitative colitis scoring. (XLSX)
