# Supplementary figures and images for: Comparison of the fecal, cecal, and mucus microbiome in male and female mice after TNBS-induced colitis
Source: PLoS One. 2019 Nov 8;14(11):e0225079. doi: 10.1371/journal.pone.0225079 (PMC6839838; doi:10.1371/journal.pone.0225079)

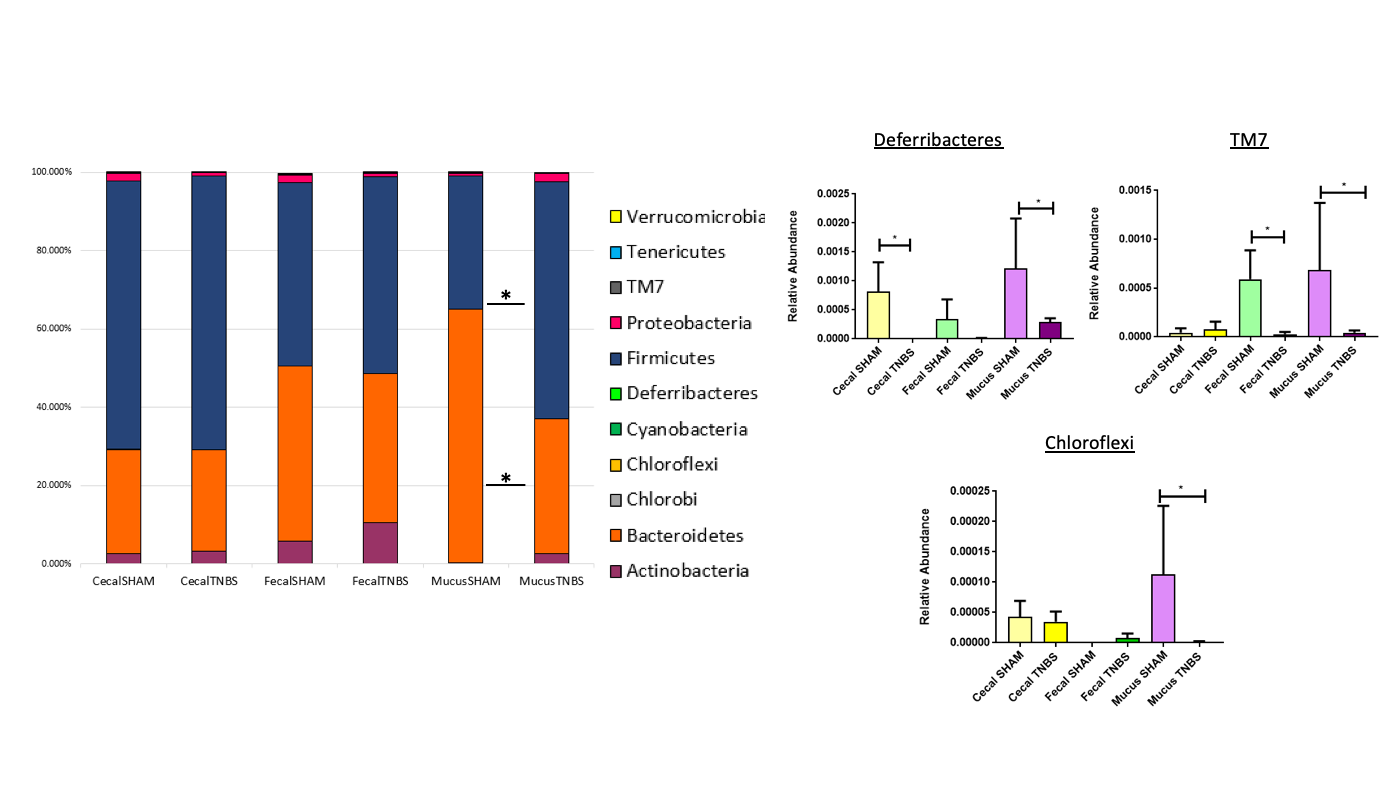

Supplement: S1 Fig — Significance indicated by asterisk. Significance found using Kruskal-Wallis with pairwise follow-up tests. P values corrected for multiple comparisons with Dunn’s test. Low abundance phyla plotted individually for visibility. (TIF) [file pone.0225079.s001.tif]
